# Supplementary material for: Different Oxidation Pathways of 2-Selenouracil and 2-Thiouracil, Natural Components of Transfer RNA
Source: Int J Mol Sci. 2020 Aug 19;21(17):5956. doi: 10.3390/ijms21175956 (PMC7503825; doi:10.3390/ijms21175956)
Supplement: Supplementary file 1 [file ijms-21-05956-s001.pdf]

**SUPPLEMENTARY MATERIALS**

# Different oxidation pathways of 2-selenouracil and 2-thiouracil, natural components of transfer RNAs

**Katarzyna Kulik,[1,&] Klaudia Sadowska,[2,&] Ewelina Wielgus,[1] Barbara Pacholczyk- Sienicka,[2] Elżbieta Sochacka,[2] Barbara Nawrot[1]***

[1]*Centre of Molecular and Macromolecular Studies, Polish Academy of Sciences, Sienkiewicza 112, 90-363 Lodz, Poland;*

[2]*Institute of Organic Chemistry, Lodz University of Technology, Zeromskiego 116, 90-924 Lodz, Poland;*

* - corresponding author bnawrot@cbmm.lodz.pl
& -equal first authorship

**Table of contents:**

1. **Chemistry**
   Synthesis of 2-selenouracil (Se2Ura**, 1a**)

2. **Spectral and mass spectrometry analysis of products**
   **Figure S1**. $^1$H NMR spectrum of **1a**
   **Figure S2**. ESI(−)-HRMS analysis and UV spectrum of **1a**
   **Figure S3**. ESI(−)-HRMS analysis and UV spectrum of **2a**
   **Figure S4**. ESI(−)-HRMS analysis and UV spectrum of **3a**
   **Figure S5**. ESI(−)-HRMS analysis of **4a** , n=1
   **Figure S6.** ESI(−)-HRMS analysis and UV spectrum of **4a ,** (n=2)
   **Figure S7.** ESI(−)-HRMS analysis and UV spectrum of **6**
   **Figure S8**. ESI(−)-HRMS analysis and UV spectrum of **7a**
   **Figure S9**. NMR analysis of **8**: (A) $^1$H NMR, (B) $^{13}$C NMR, (C) COSY, (D) HMQC
   **Figure S10.** ESI(−)-HRMS analysis and UV spectrum of **8**
   **Figure S11** . NMR analysis of **9**: (A) $^1$H NMR, (B) $^{13}$C NMR, (C) COSY, (D) HMQC
   **Figure S12**. ESI(−)-HRMS analysis and UV spectrum of **9**
   **Figure S13.** The pH-dependent UV spectra of **9** in the pH range from 3 to 10
   **Figure S14**. ESI(−)-HRMS analysis and UV spectrum of **2b**
   **Figure S15**. ESI(−)-HRMS analysis of **3b**
   **Figure S16**. ESI(−)-HRMS analysis of **4b**, (n=1)
   **Figure S17**. ESI(−)-HRMS analysis of **4b**, (n=2)
   **Figure S18**. ESI(−)-HRMS analysis and UV spectrum of **4b**, (n=3)
   **Figure S19**. ESI(−)-HRMS analysis of **7b**
   **Figure S20**. ESI(−)-HRMS analysis and UV spectrum of **10**

3. **Time course of the oxidation of Se2Ura (1a) by hydrogen peroxide monitored by $^1$H NMR spectroscopy and UPLC-PDA-ESI(−)-HRMS**

   **Figure S21.** $^1$H NMR analysis of the reaction mixtures for oxidation of Se2Ura (**1a**, 10 mM) with $H_2O_2$ (10 mM) in 67 mM phosphate buffer pH 8.0, at room temperature.

**FigureS22.** $^1$H NMR analysis of the reaction mixtures for oxidation of Se2Ura (**1a**, 10 mM) with $H_2O_2$ (10 mM) in 67 mM phosphate buffer pH 5.0, at room temperature.
**Figure S23.** $^1$H NMR analysis of the reaction mixtures for oxidation of Se2Ura (**1a**, 10 mM) with $H_2O_2$ (10 mM) in water, at room temperature.
**Figure S24.** $^1$H NMR analysis of the reaction mixtures for oxidation of Se2Ura (**1a**, 10 mM) with $H_2O_2$ (5 mM) in 67 mM phosphate buffer pH 7.4, at room temperature.
**Figure S25.** $^1$H NMR analysis of the reaction mixtures for oxidation of Se2Ura (**1a**, 10 mM) with $H_2O_2$ (100 mM) in 67 mM phosphate buffer pH 7.4, at room temperature.
**Figure S26**. UPLC-PDA chromatographic analysis of the reaction mixtures for oxidation of Se2Ura (**1a**, 10 mM) with $H_2O_2$ (100 mM) in 67 mM phosphate buffer pH 7.4 , at room temperature.

4. **MS/MS fragmentation**
**Figure S27**. Product ion mass spectrum of **10**.

5. **Time course of the oxidation of S2Ura (1b) by hydrogen peroxide monitored by $^1$H NMR spectroscopy and UPLC-PDA-ESI(−)-HRMS**

**Figure S28.** The time course of the formation of products for the oxidation reaction of S2Ura (**1b**, 10 mM) with $H_2O_2$ (10 mM) in 67 mM phosphate buffer pH 7.4, r.t.
**Figure S29.** $^1$H NMR analysis of the reaction mixtures for oxidation of S2Ura (**1b**, 10 mM) with $H_2O_2$ (100 mM) in 67 mM phosphate buffer pH 7.4, at room temperature (r.t.).

6. **Mechanism of formation of 9.**
**Fig. S30.** $^1$H NMR analysis (H5 and H6) of the reaction of **8** (4 mM) with **1a** (10 mM) at the 0.4:1 molar ratio, at 67 mM phosphate buffer pH 8, r.t. Traces of compound **9** are seen after 17 and 24 h (signals of H5 and H6 protons are indicated by arrows).
**Fig. S31**. $^1$H NMR analysis (H5 and H6) of the reaction of **8** (4 mM) and **1a** (10 mM) at the 0.4:1 molar ratio, after addition of 0.5 eq. of $H_2O_2$ (5 mM), at 67 mM phosphate buffer pH 8, r.t. Signals of compound **9** are seen after 1 h (see signals of H5 and H6 protons indicated by arrows).

## 1. Synthesis of 2-selenouracil (Se2Ura, 1a)

Sodium pieces (5 g, 0.217 mol) were suspended in dry toluene (27 mL) under inert atmosphere in three necked flask (equipped with reflux condenser and a $CaCl_2$ tube) and heated at 120°C until the sodium melted. Then ethanol was added (15.2 mL, 0.26 mol), and the reaction was carried out for 3 hours. After cooling, 136 mL of diethyl ether were added, followed by the dropwise addition of ethyl acetate (19.2 mL 0.197 mol) and ethyl formate (15.9 mL 0.197 mol) and stirred for 24 hours. The solvent was evaporated under reduced pressure and the crude product was used for the next reaction.
Freshly prepared, crude ethyl 3-oxopropanoate (2.7 g, 19.6 mmol) was dissolved in 10 mL of ethanol and heated at 70°C, then selenourea (1 g, 8.13 mmol) was added and reaction was heated for 3 hours. The reaction was cooled, the red selenium residue was removed by filtration and the remaining solution was neutralized with 5% HCl. The solvent was evaporated under reduced pressure. Crystallization from ethanol yielded 697 mg of **1a** (49%). Spectral data are given Fig. S1 and S2. $^1$H NMR (700 MHz, $D_2O$) δ (ppm): 6.12 (d, 1H, *J*=7.7), 7.61 (d, 1H, J=7.7).

## 2. Spectral and mass spectrometry analysis of products

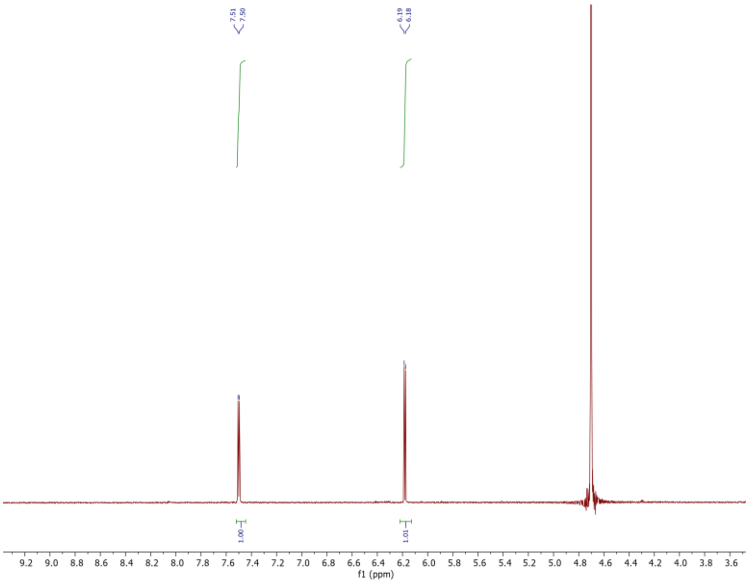


**Figure S1**. $^{1}$H NMR spectrum of **1a**

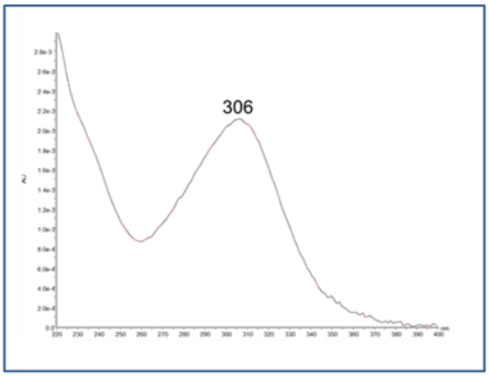


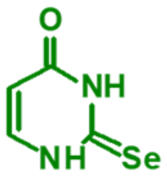


Exact mass = 175.9489

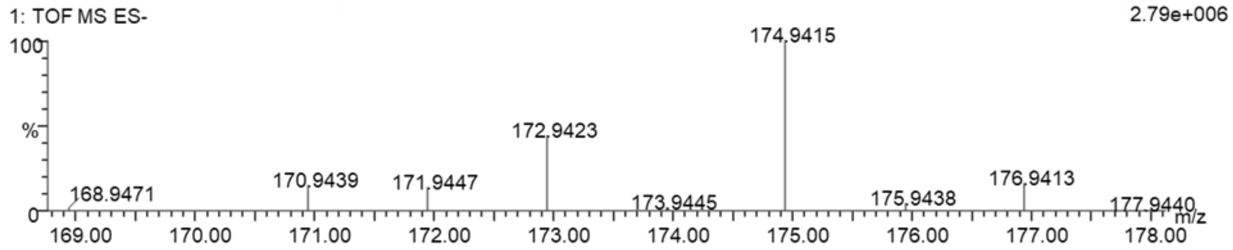


| | | | | | | | | |
|---|---|---|---|---|---|---|---|---|
| Minimum: | | | | -1.5 | | | | |
| Maximum: | | 5.0 | 10.0 | 70.0 | | | | |
| Mass | Calc. Mass | mDa | PPM | DBE | i-FIT | Norm | Conf(%) | Formula |
| 174.9415 | 174.9411 | 0.4 | 2.3 | 5.5 | 43.7 | n/a | n/a | C4 H3 N2 O Se |

**Figure S2.** ESI(−)-HRMS analysis and UV spectrum of **1a**

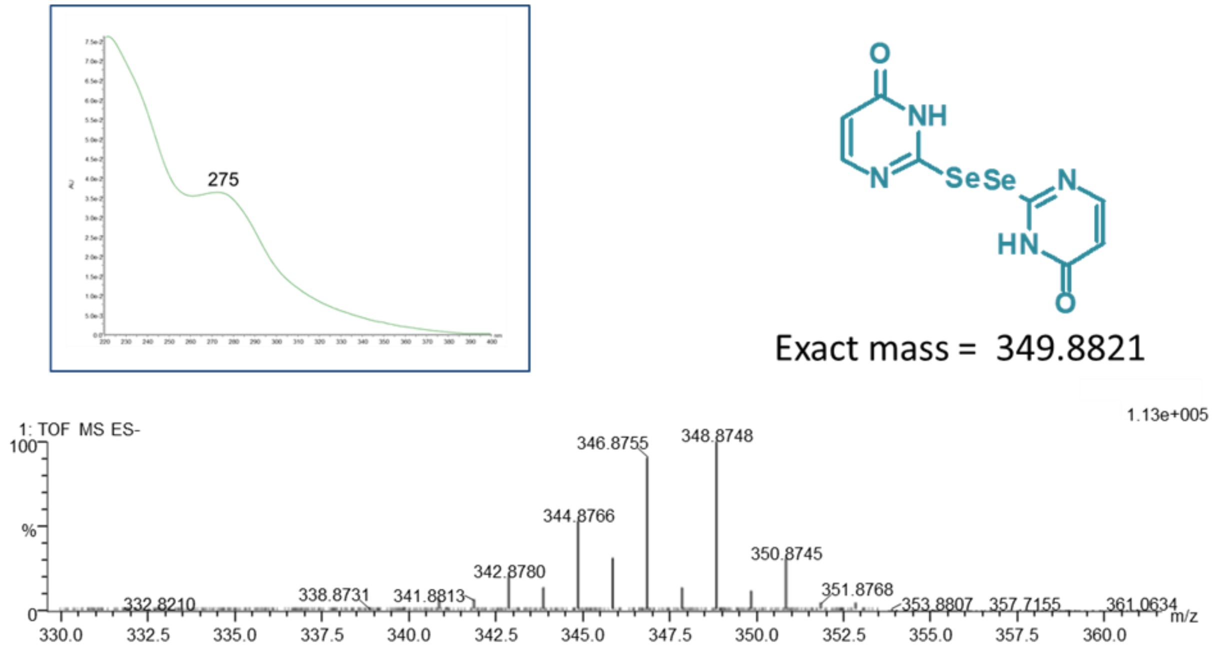


| | Calc. Mass | mDa | PPM | DBE | i-FIT | Norm | Conf(%) | Formula |
|---|---|---|---|---|---|---|---|---|
| Minimum: | | | | -1.5 | | | | |
| Maximum: | | 5.0 | 5.0 | 70.0 | | | | |

| Mass | Calc. Mass | mDa | PPM | DBE | i-FIT | Norm | Conf(%) | Formula |
|---|---|---|---|---|---|---|---|---|
| 348.8748 | 348.8743 | 0.5 | 1.4 | 10.5 | 1060.3 | n/a | n/a | C8 H5 N4 O2 Se2 |

**Figure S3.** ESI(−)-HRMS analysis and UV spectrum of **2a**

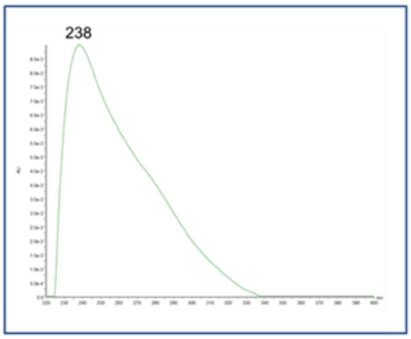


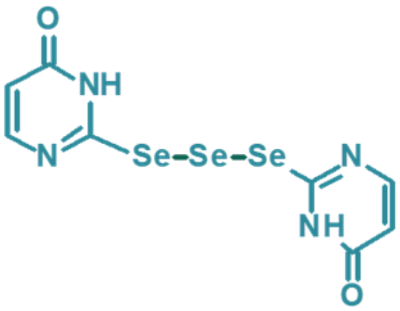


Exact mass = 429.7986

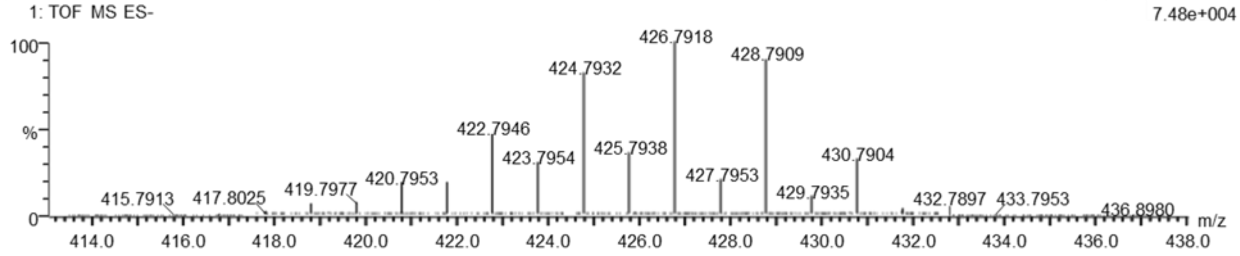


| | | | | | | | | |
|---|---|---|---|---|---|---|---|---|
| Minimum: | | | | -1.5 | | | | |
| Maximum: | | 5.0 | 5.0 | 70.0 | | | | |
| Mass | Calc. Mass | mDa | PPM | DBE | i-FIT | Norm | Conf(%) | Formula |
| 428.7909 | 428.7908 | 0.1 | 0.2 | 11.5 | 1294.1 | n/a | n/a | C8 H5 N4 O2 Se3 |

**Figure S4**. ESI(−)-HRMS analysis and UV spectrum of **3a** .

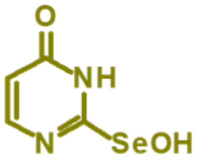


Exact mass = 191.9438

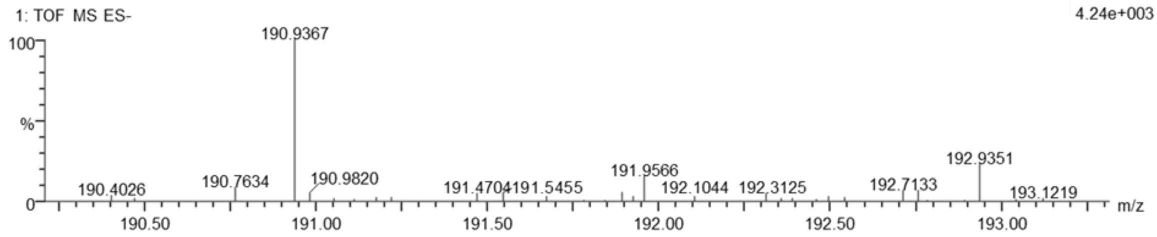


| | | | | | | | | |
|---|---|---|---|---|---|---|---|---|
| Minimum: | | | | -1.5 | | | | |
| Maximum: | | 5.0 | 10.0 | 70.0 | | | | |
| Mass | Calc. Mass | mDa | PPM | DBE | i-FIT | Norm | Conf(%) | Formula |
| 190.9367 | 190.9360 | 0.7 | 3.7 | 5.5 | 238.6 | n/a | n/a | C4 H3 N2 O2 Se |

**Figure S5.** ESI(−)-HRMS analysis of **4a**, n=1

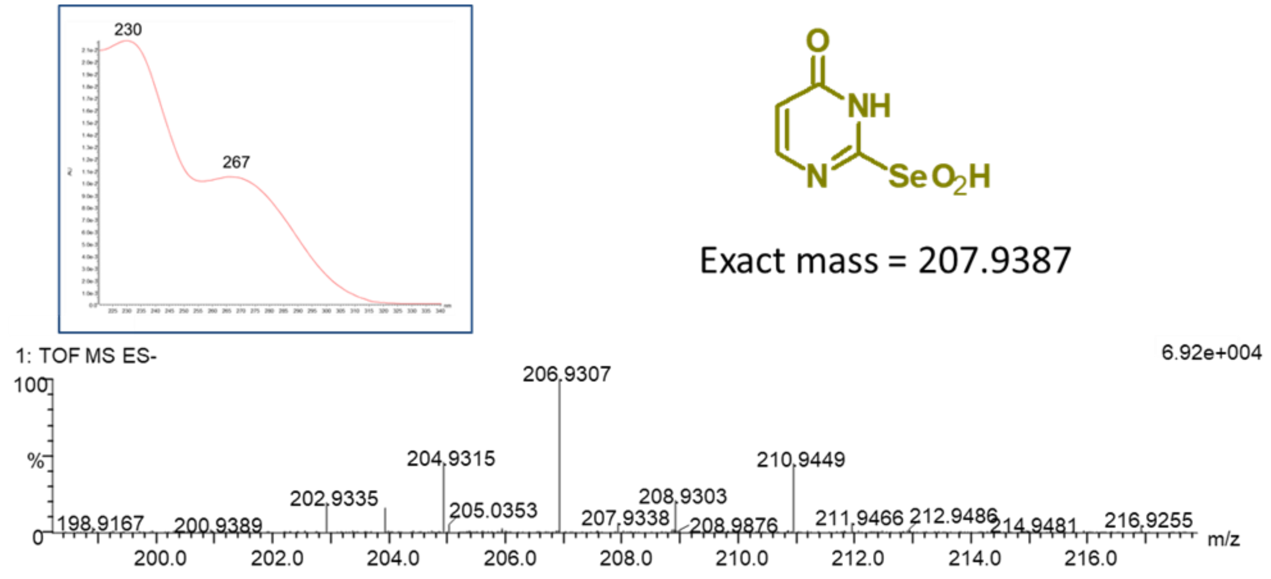


| Minimum: | | | | -10.0 | | | | |
|---|---|---|---|---|---|---|---|---|
| Maximum: | | 15.0 | 5.0 | 60.0 | | | | |
| Mass | Calc. Mass | mDa | PPM | DBE | i-FIT | Norm | Conf(%) | Formula |
| 206.9307 | 206.9309 | -0.2 | -1.0 | 5.5 | 681.3 | n/a | n/a | C4 H3 N2 O3 Se |

**Figure S6.** ESI(−)-HRMS analysis and UV spectrum of **4a**, (n=2)

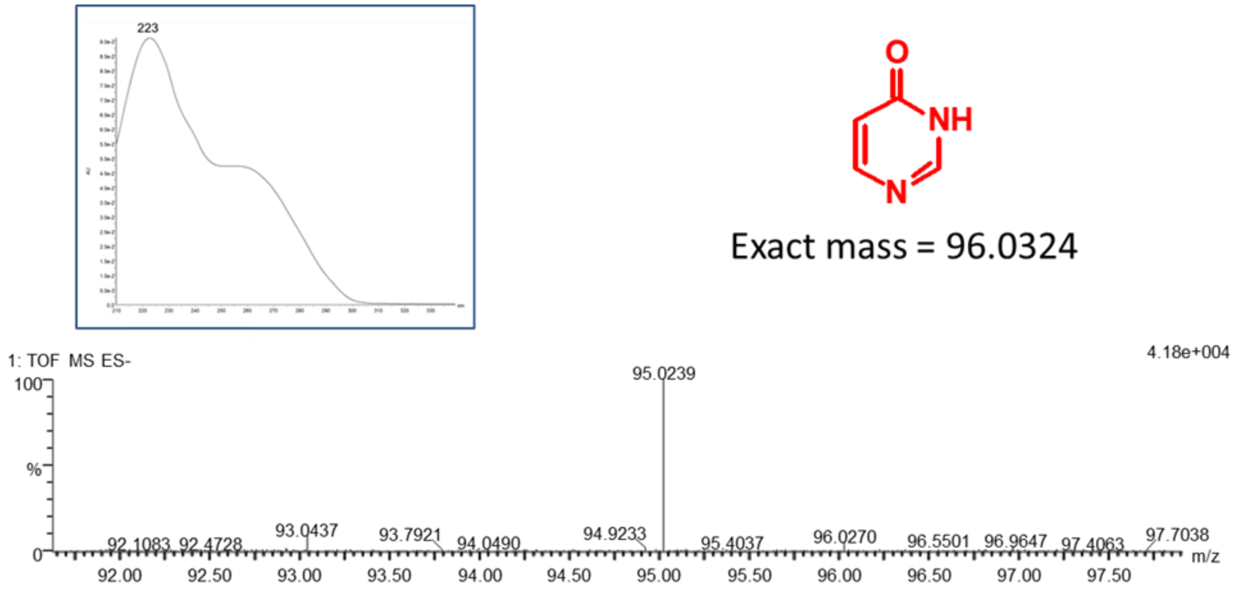


| Minimum: | | | | -1.5 | | | | |
|---|---|---|---|---|---|---|---|---|
| Maximum: | | 5.0 | 10.0 | 70.0 | | | | |
| Mass | Calc. Mass | mDa | PPM | DBE | i-FIT | Norm | Conf(%) | Formula |
| 95.0239 | 95.0245 | -0.6 | -6.3 | 4.5 | 878.3 | n/a | n/a | C4 H3 N2 O |

**Figure S7**. ESI(−)-HRMS analysis and UV spectrum of **6**

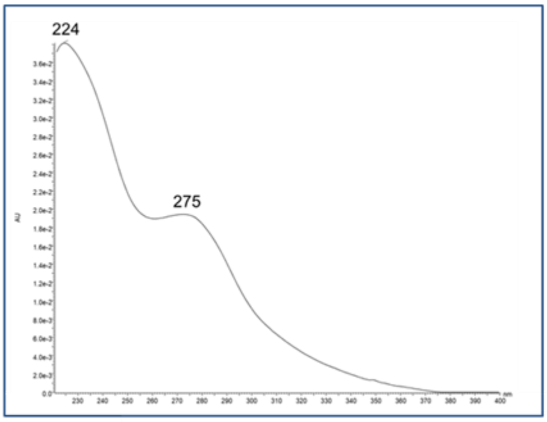


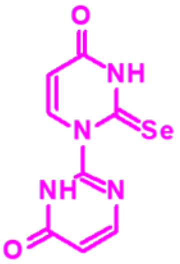


Exact mass = 269.9656

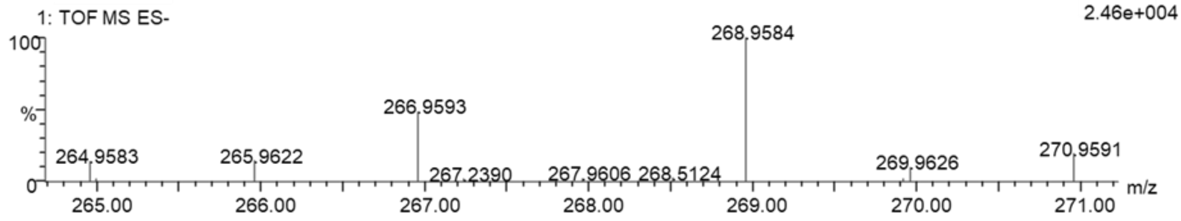


| | | | | | | | | |
|---|---|---|---|---|---|---|---|---|
| Minimum: | | | | -10.0 | | | | |
| Maximum: | | 15.0 | 15.0 | 60.0 | | | | |
| Mass | Calc. Mass | mDa | PPM | DBE | i-FIT | Norm | Conf(%) | Formula |
| 268.9584 | 268.9578 | 0.6 | 2.2 | 9.5 | 77.3 | 0.695 | 49.89 | C8 H5 N4 O2 Se |

**Figure S8.** ESI(−)-HRMS analysis and UV spectrum of **7a.**

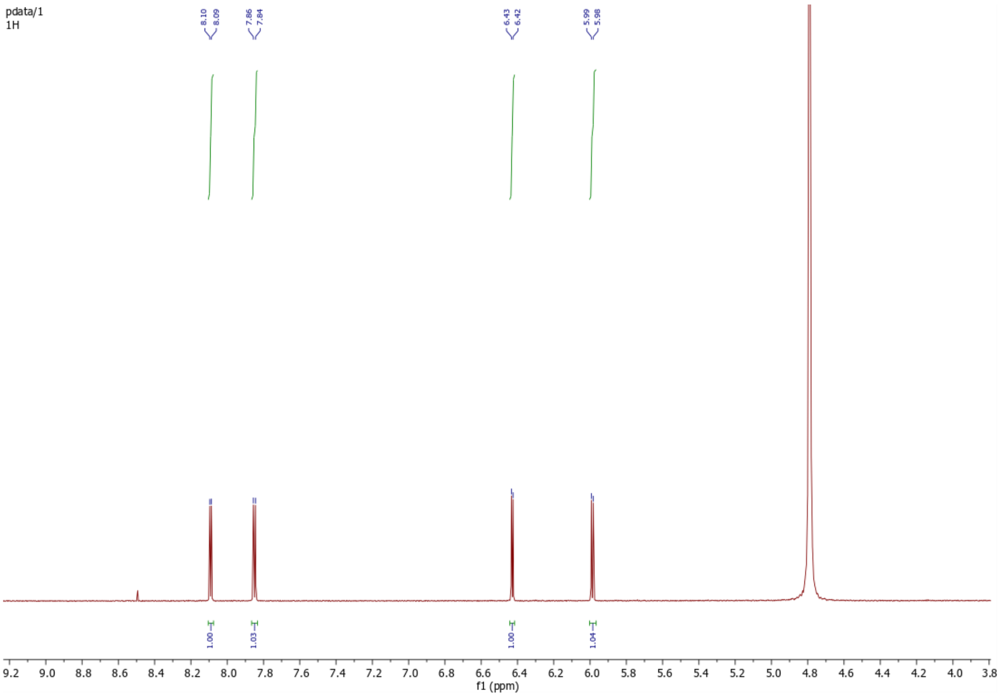


**Figure S9_A.** $^{1}H$ NMR (700 MHz, $D_2O$) spectrum of the **8**.

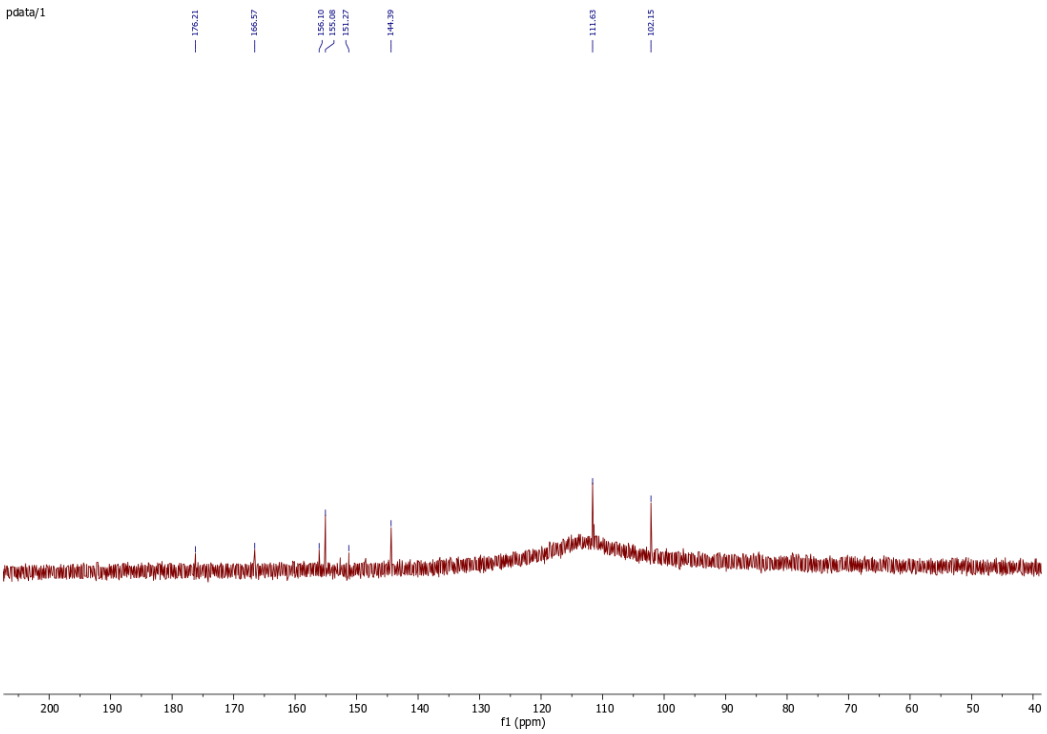


**Figure S9_B.** $^{13}$C NMR (176 MHz, $D_2O$) spectrum of the **8**.

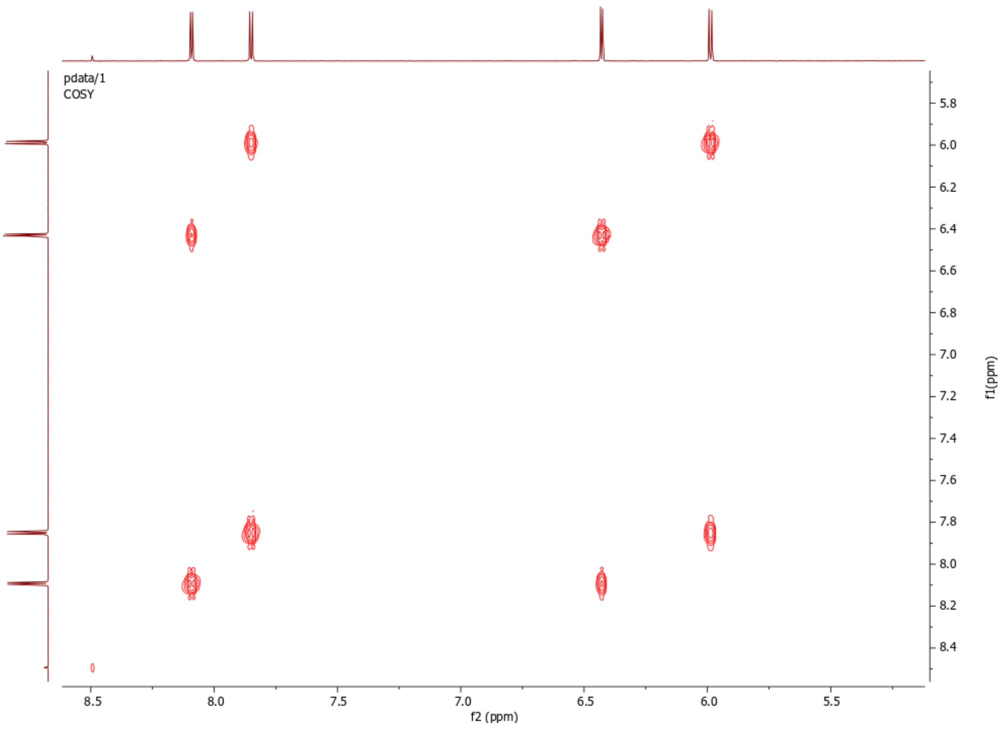


**Figure S9_C.** $^{13}$COSY (700 MHz, $D_2O$) of **8**.

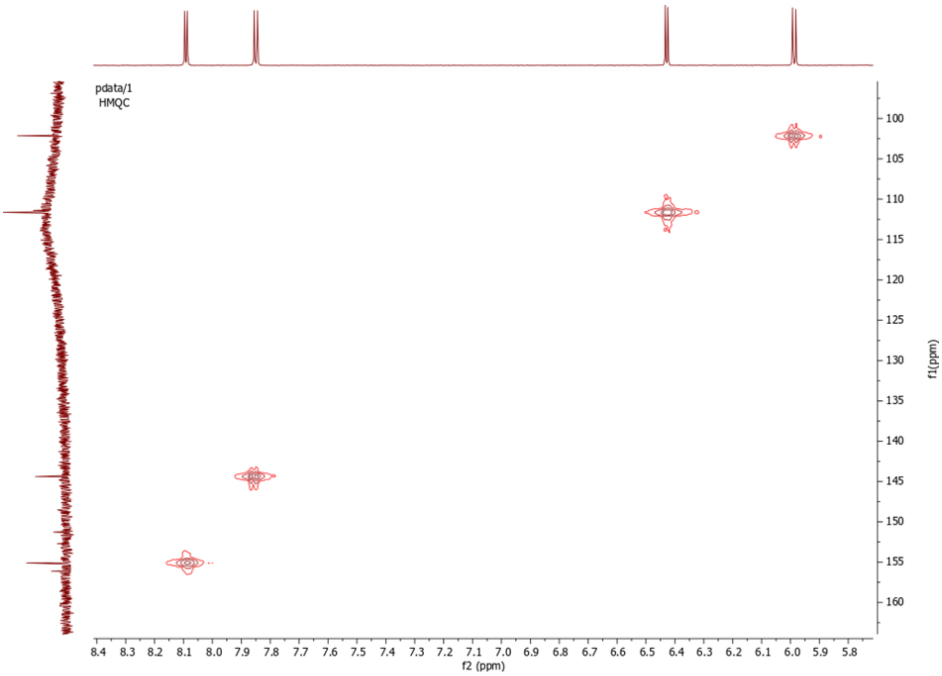


**Figure S9_D.** HMQC (700 MHz, $D_2O$) of **8**.

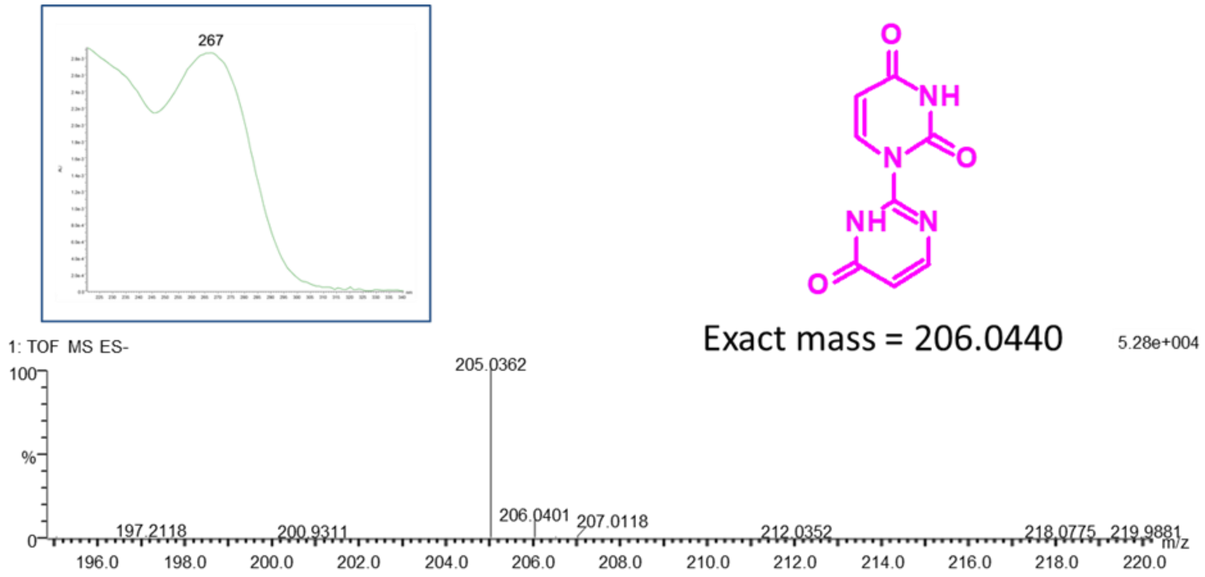


| | Mass | Calc. Mass | mDa | PPM | DBE | i-FIT | Norm | Conf(%) | Formula |
|---|---|---|---|---|---|---|---|---|---|
| Minimum: | | | | | -1.5 | | | | |
| Maximum: | | | 5.0 | 10.0 | 70.0 | | | | |
| | 205.0362 | 205.0362 | 0.0 | 0.0 | 8.5 | 63.0 | n/a | n/a | C8 H5 N4 O3 |

**Figure S10**. ESI(−)-HRMS analysis and UV spectrum of **8**

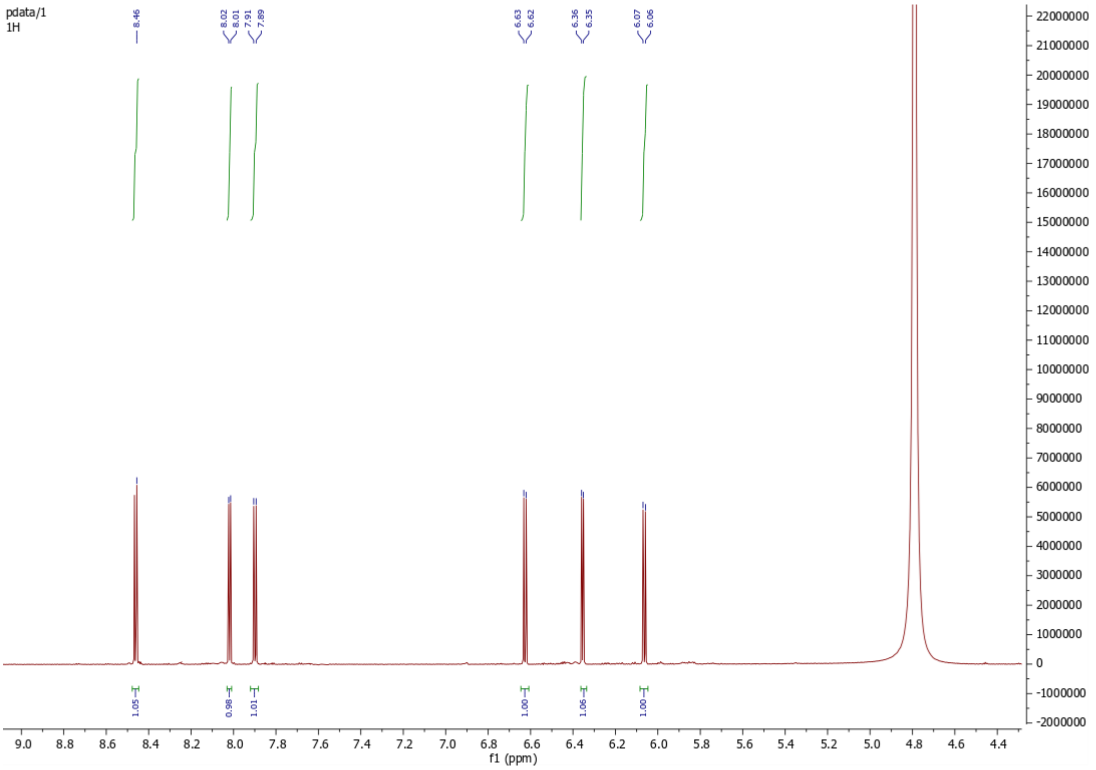

**Figure S11_A.** $^{1}H$ NMR (700 MHz, $D_2O$) spectrum of the **9**.

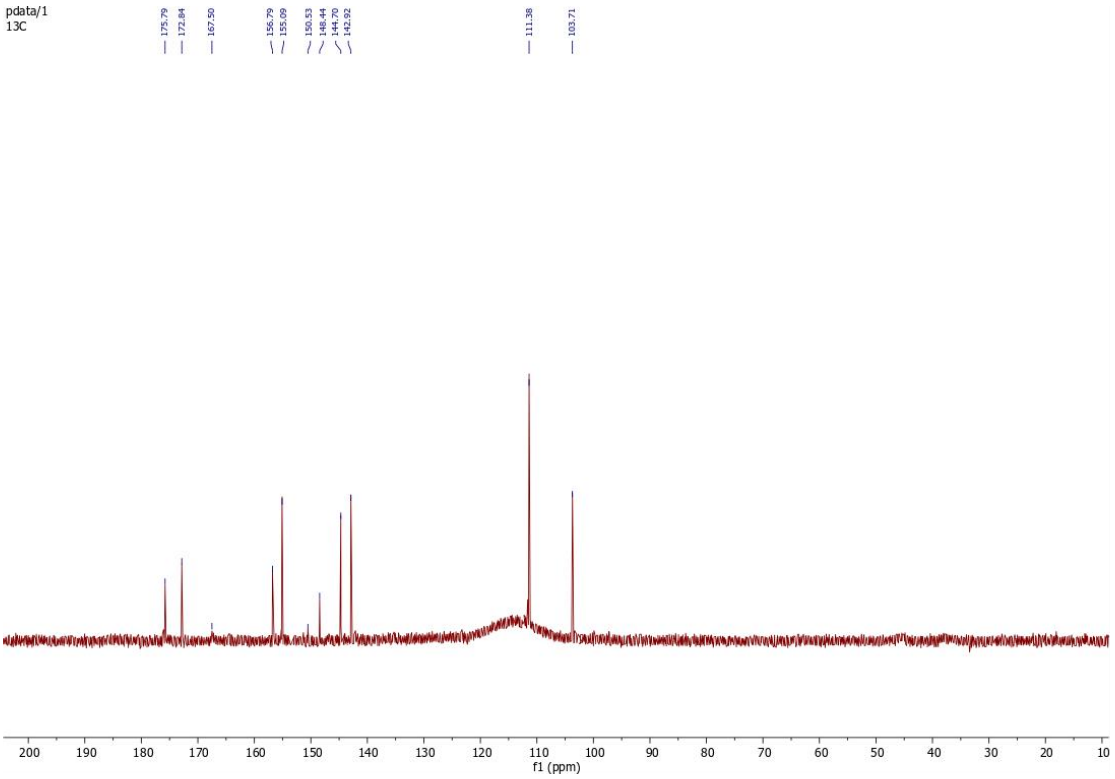

**Figure S11_B.** $^{13}C$ NMR (176 MHz, $D_2O$) spectrum of the **9**.

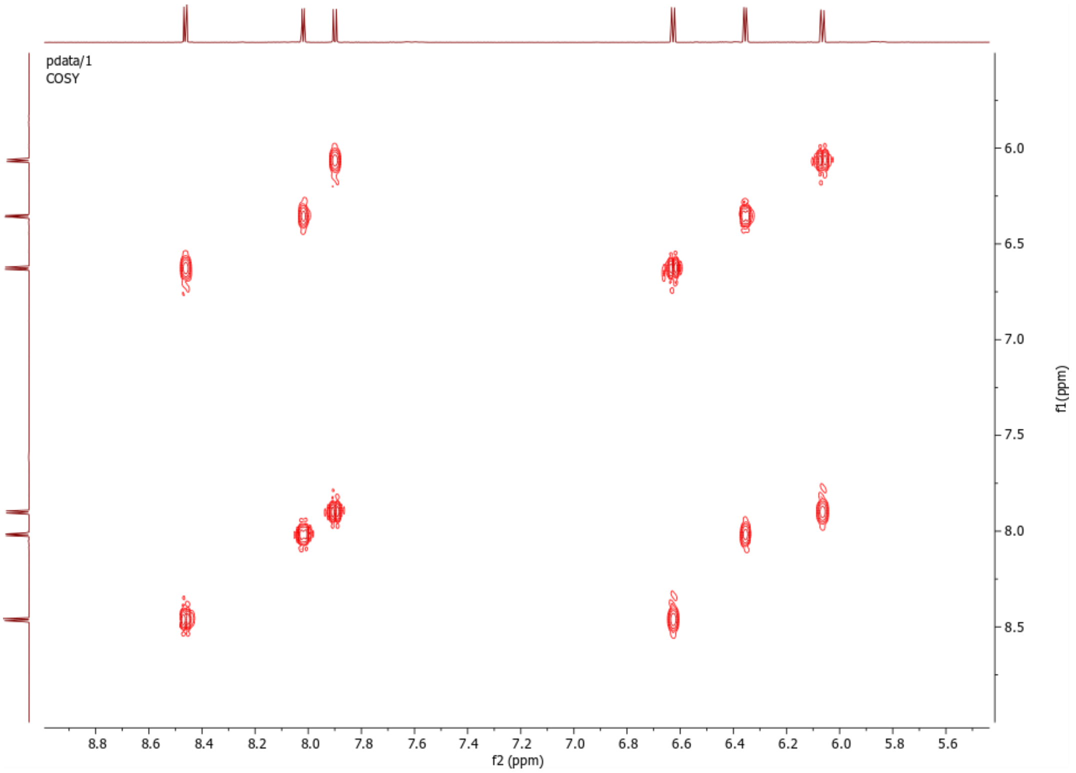


**Figure S11_C.** $^{13}$COSY (700 MHz, $D_2O$) of **9**.

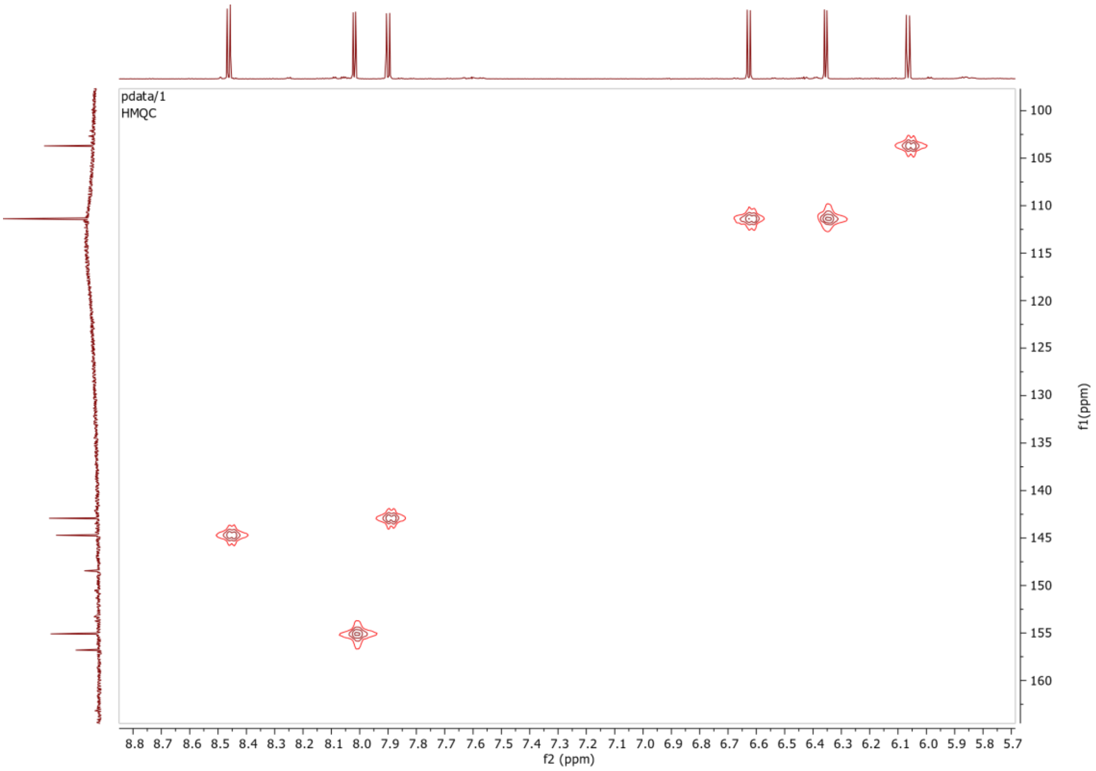


**Figure S11_D.** HMQC (700 MHz, $D_2O$) of **9**.

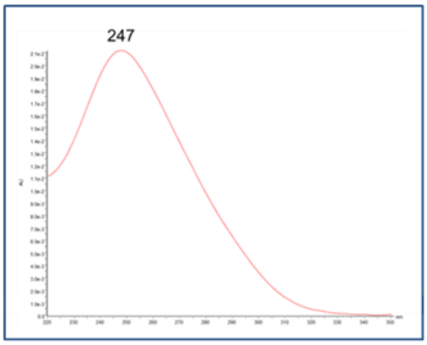


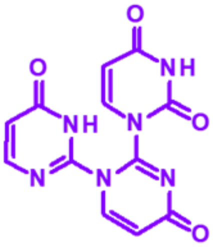


Exact mass = 300.0607

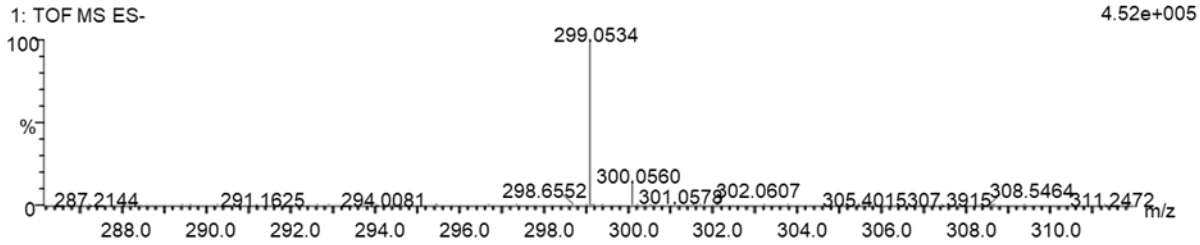


| | | | | |
|---|---|---|---|---|
| Minimum: | | | | -1.5 |
| Maximum: | | 5.0 | 5.0 | 70.0 |

| Mass | Calc. Mass | mDa | PPM | DBE | i-FIT | Norm | Conf(%) | Formula |
|---|---|---|---|---|---|---|---|---|
| 299.0534 | 299.0529 | 0.5 | 1.7 | 12.5 | 184.3 | 0.637 | 52.89 | C12 H7 N6 O4 |

**Figure S12.** ESI(−)-HRMS analysis and UV spectrum of **9.**

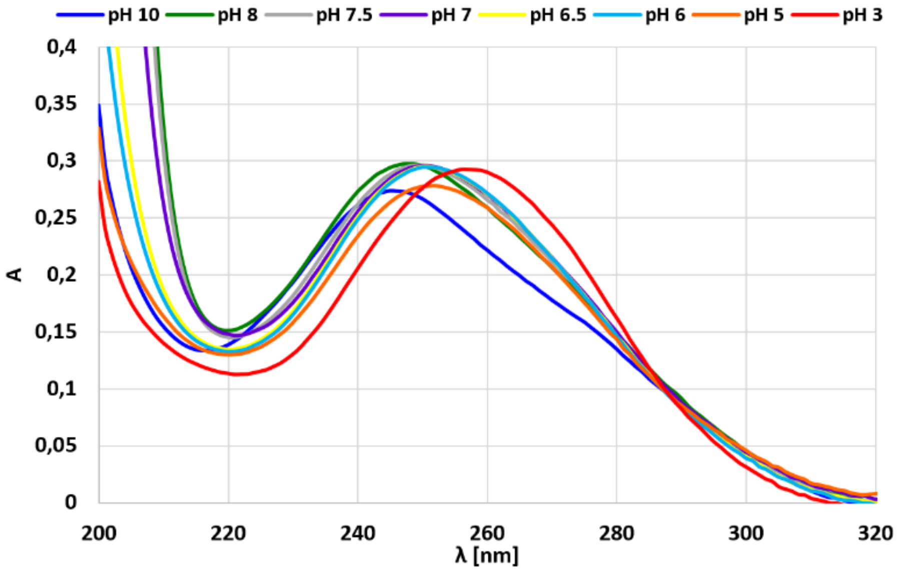


**Figure S13.** The pH-dependent UV spectra of **9** in the pH range from 3 to 10.

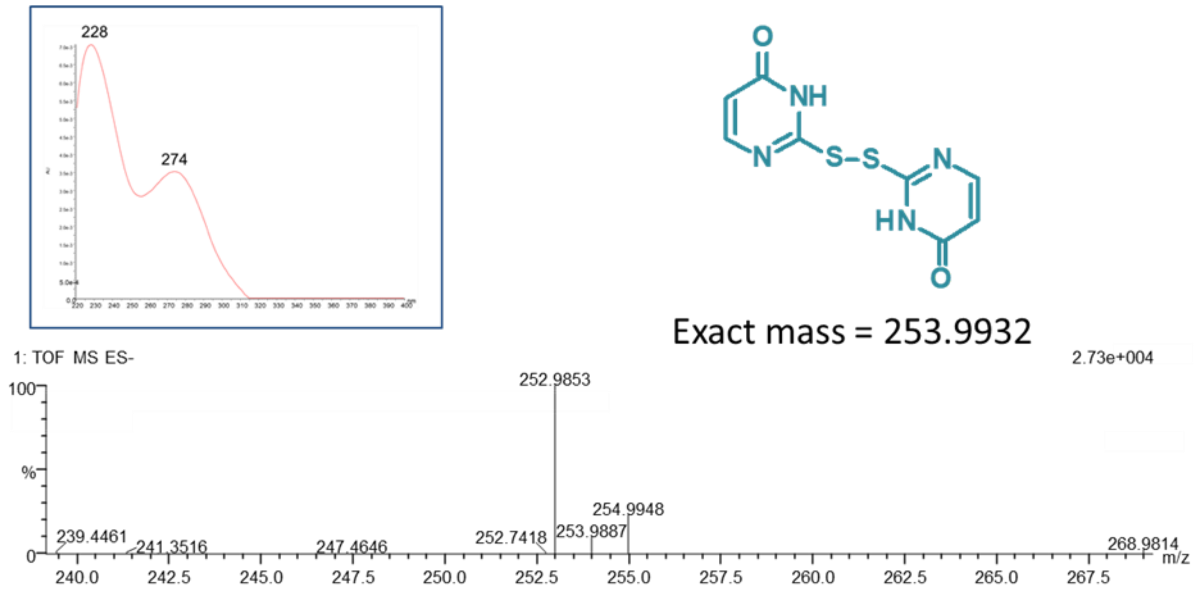


| | Mass | Calc. Mass | mDa | PPM | DBE | i-FIT | Norm | Conf(%) | Formula |
|---|---|---|---|---|---|---|---|---|---|
| Minimum: | | | | | -1.5 | | | | |
| Maximum: | | | 5.0 | 10.0 | 70.0 | | | | |
| | 252.9853 | 252.9854 | -0.1 | -0.4 | 8.5 | 67.8 | 11.794 | 0.00 | C8 H5 N4 O2 S2 |

**Figure S14**. ESI(−)-HRMS analysis and UV spectrum of **2b.**

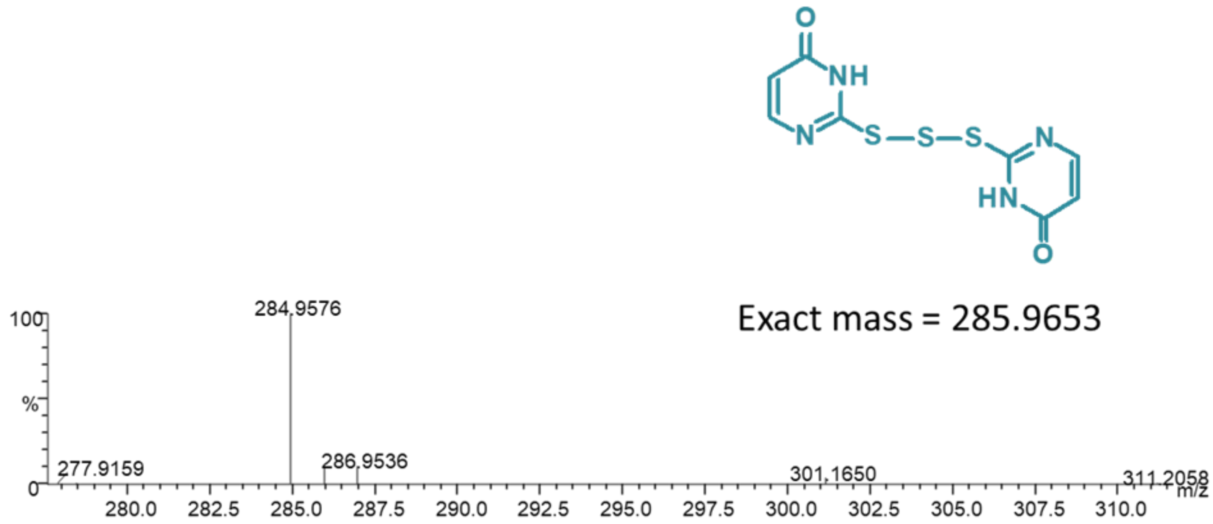


| | Mass | Calc. Mass | mDa | PPM | DBE | i-FIT | Norm | Conf(%) | Formula |
|---|---|---|---|---|---|---|---|---|---|
| Minimum: | | | | | -1.5 | | | | |
| Maximum: | | | 5.0 | 5.0 | 70.0 | | | | |
| | 284.9576 | 284.9575 | 0.1 | 0.4 | 8.5 | 64.5 | 0.047 | 95.41 | C8 H5 N4 O2 S3 |

**Figure S15**. ESI(−)-HRMS analysis of **3b.**

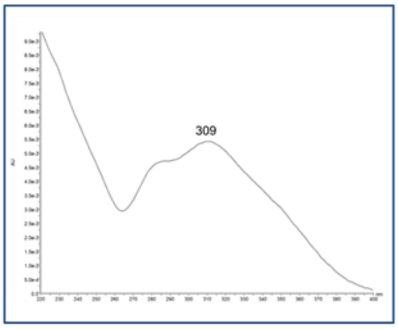


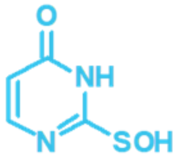


Exact mass = 143.9994

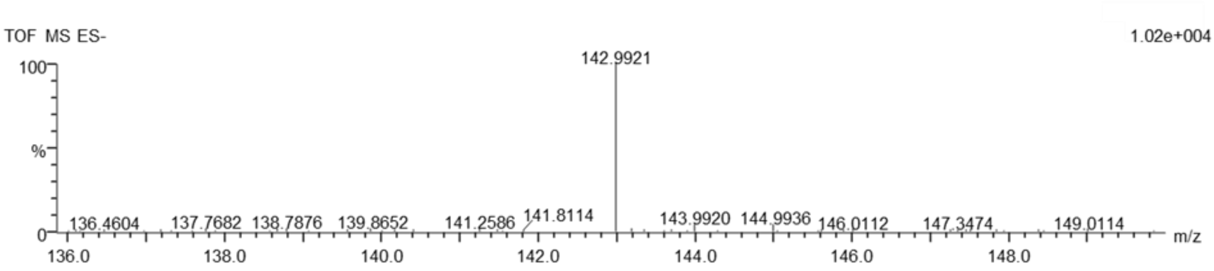


| | | | | | | | | |
|---|---|---|---|---|---|---|---|---|
| Minimum: | | | | -1.5 | | | | |
| Maximum: | | 5.0 | 10.0 | 70.0 | | | | |
| Mass | Calc. Mass | mDa | PPM | DBE | i-FIT | Norm | Conf(%) | Formula |
| 142.9921 | 142.9915 | 0.6 | 4.2 | 4.5 | 190.0 | n/a | n/a | C4 H3 N2 O2 S |

**Figure S16**. ESI(−)-HRMS analysis of **4b**, (n=1)

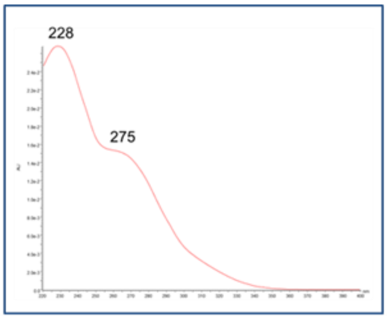


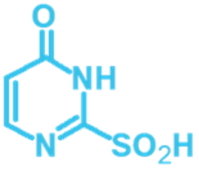


Exact mass = 159.9943

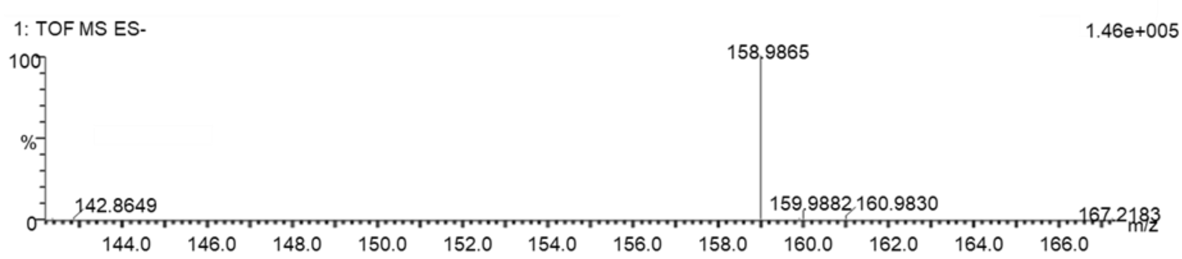


| | | | | | | | | |
|---|---|---|---|---|---|---|---|---|
| Minimum: | | | | -1.5 | | | | |
| Maximum: | | 5.0 | 5.0 | 70.0 | | | | |
| Mass | Calc. Mass | mDa | PPM | DBE | i-FIT | Norm | Conf(%) | Formula |
| 158.9865 | 158.9864 | 0.1 | 0.6 | 4.5 | 73.1 | n/a | n/a | C4 H3 N2 O3 S |

**Figure S17**. ESI(−)-HRMS analysis of **4b**, (n=2)

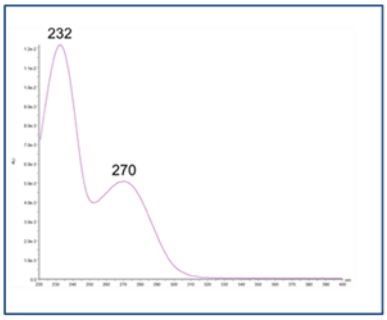


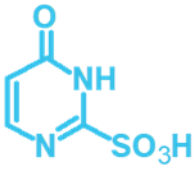


Exact mass = 175.9892

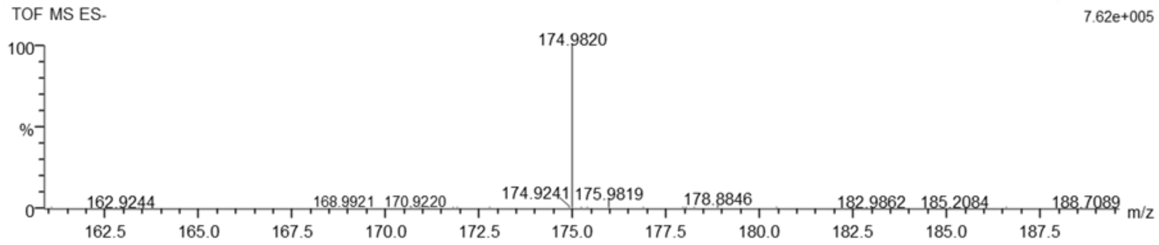


| | | | | | | | | |
|---|---|---|---|---|---|---|---|---|
| Minimum: | | | | -1.5 | | | | |
| Maximum: | | 5.0 | 10.0 | 70.0 | | | | |

| Mass | Calc. Mass | mDa | PPM | DBE | i-FIT | Norm | Conf(%) | Formula |
|---|---|---|---|---|---|---|---|---|
| 174.9820 | 174.9814 | 0.6 | 3.4 | 4.5 | 152.1 | n/a | n/a | C4 H3 N2 O4 S |

**Figure S18**. ESI(−)-HRMS analysis and UV spectrum of **4b**, (n=3)

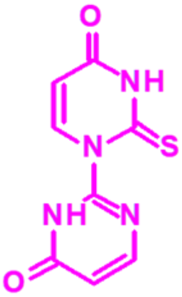


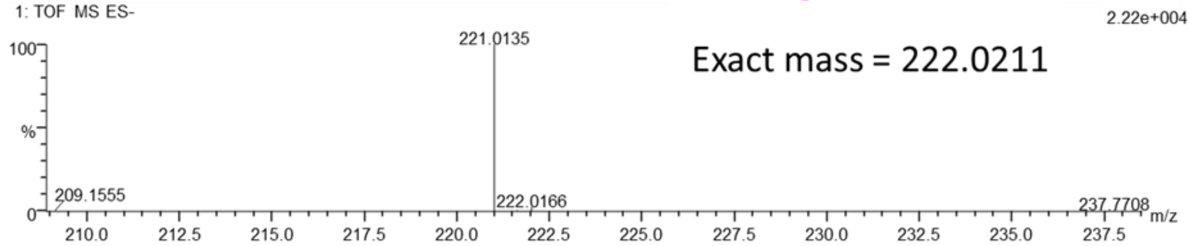


Exact mass = 222.0211

| | | | | | | | | |
|---|---|---|---|---|---|---|---|---|
| Minimum: | | | | -1.5 | | | | |
| Maximum: | | 5.0 | 10.0 | 70.0 | | | | |

| Mass | Calc. Mass | mDa | PPM | DBE | i-FIT | Norm | Conf(%) | Formula |
|---|---|---|---|---|---|---|---|---|
| 221.0135 | 221.0133 | 0.2 | 0.9 | 8.5 | 64.0 | 3.235 | 3.93 | C8 H5 N4 O2 S |

**Figure S19**. ESI(−)-HRMS analysis of **7b**

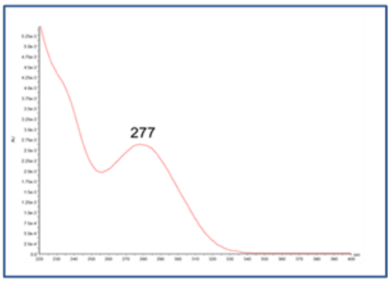


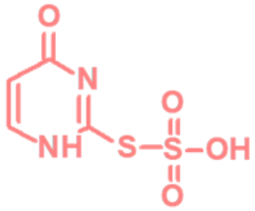


Exact mass = 207.9613

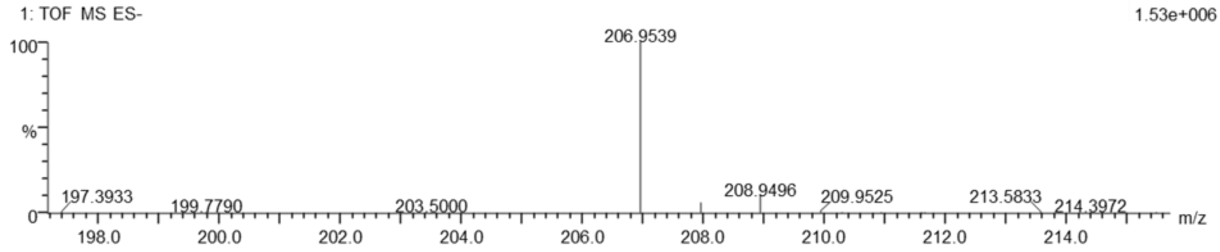


| | | | | | | | | |
|---|---|---|---|---|---|---|---|---|
| Minimum: | | | | -1.5 | | | | |
| Maximum: | | 5.0 | 10.0 | 70.0 | | | | |
| Mass | Calc. Mass | mDa | PPM | DBE | i-FIT | Norm | Conf(%) | Formula |
| 206.9539 | 206.9534 | 0.5 | 2.4 | 4.5 | 98.9 | n/a | n/a | C4 H3 N2 O4 S2 |

**Figure S20**. ESI(−)-HRMS analysis and UV spectrum of **10**.

## 3. Time course of the oxidation of Se2Ura (1a) by hydrogen peroxide monitored by $^1$H NMR spectroscopy and UPLC-PDA-ESI(−)-HRMS

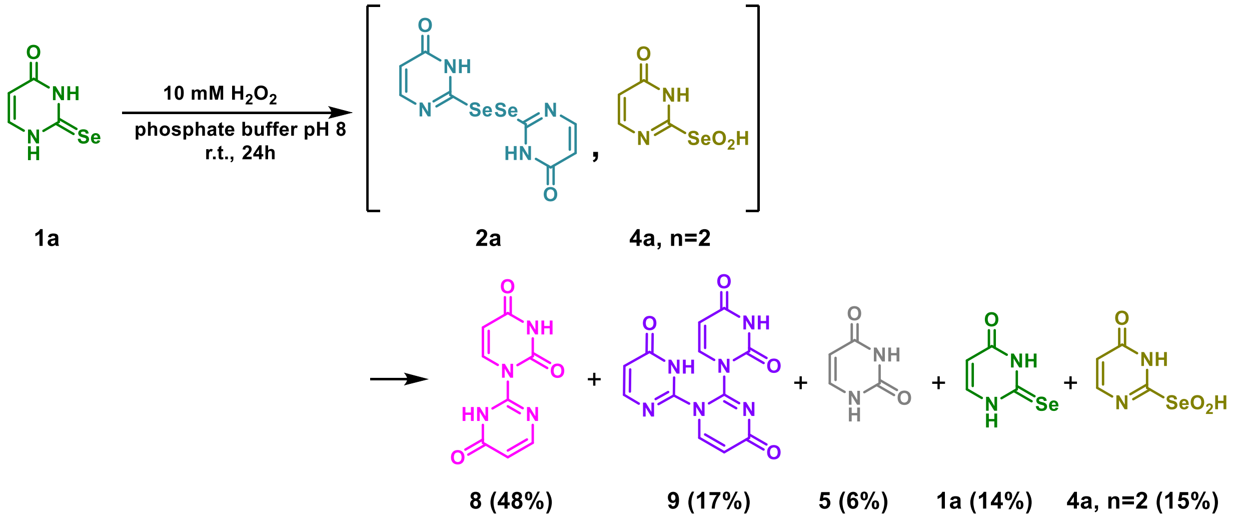

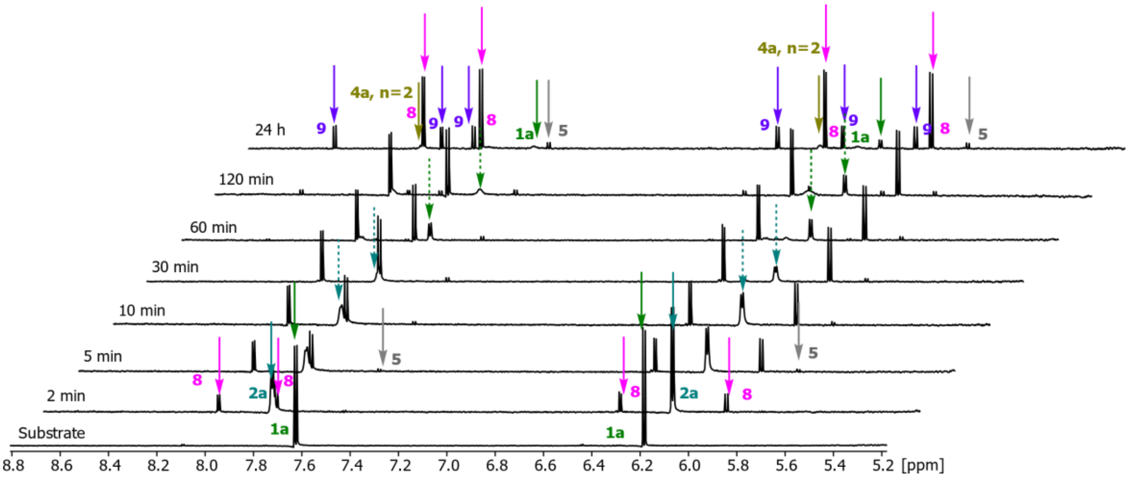


**Figure S21.** $^{1}$H NMR analysis of the reaction mixtures for oxidation of Se2Ura (**1a**, 10 mM) with $H_2O_2$ (10 mM) in 67 mM phosphate buffer pH 8.0, at room temperature

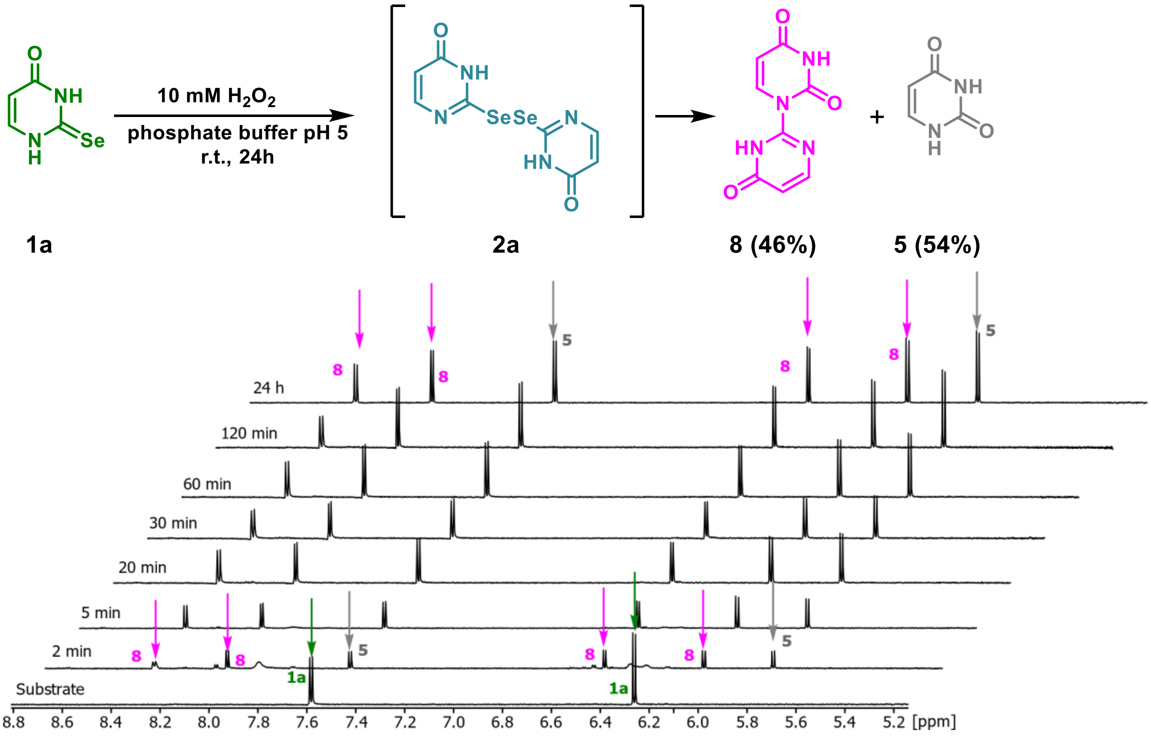


**Figure S22.** $^{1}$H NMR analysis of the reaction mixtures for oxidation of Se2Ura (**1a**, 10 mM) with $H_2O_2$ (10 mM) in 67 mM phosphate buffer pH 5.0, at room temperature.

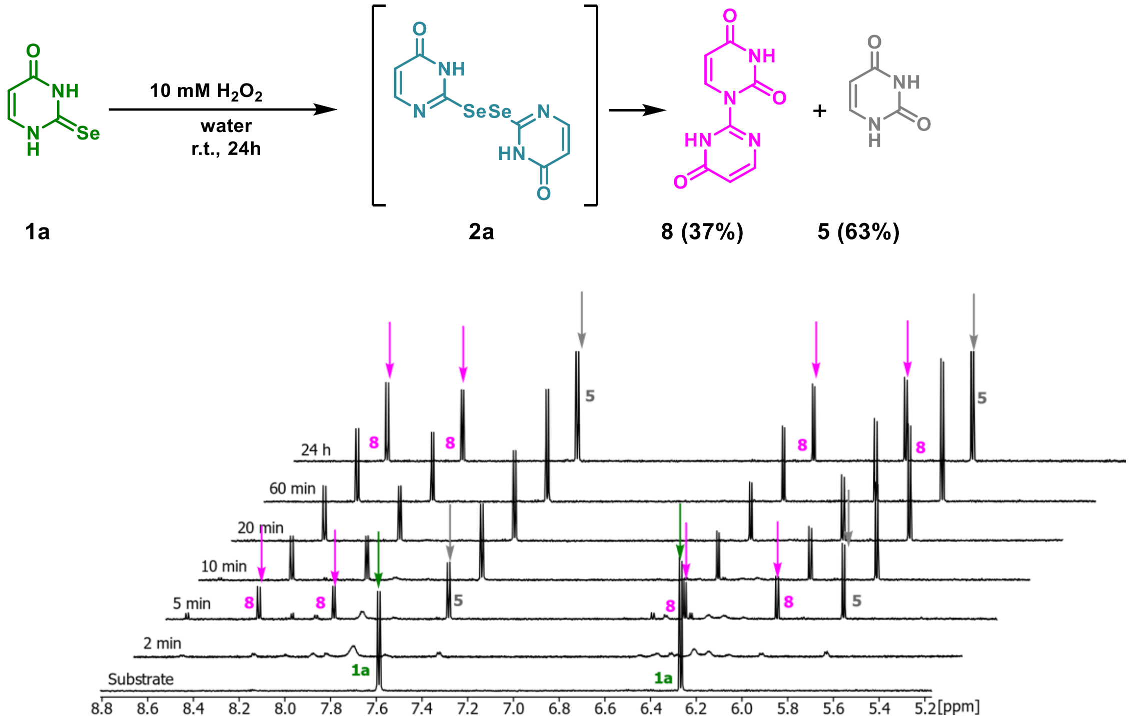


**Figure S23.** $^{1}$H NMR analysis of the reaction mixtures for oxidation of Se2Ura (**1a**, 10 mM) with $H_2O_2$ (10 mM) in water, at room temperature

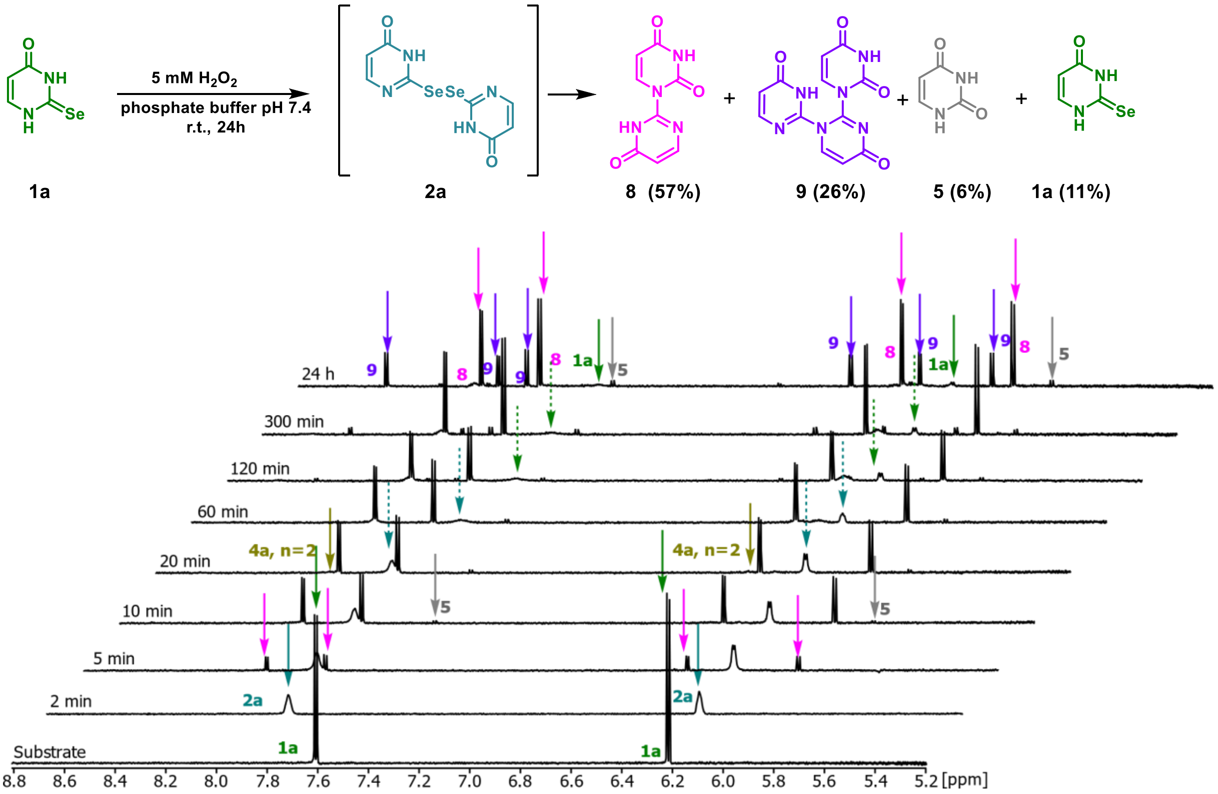


**Figure S24.** $^{1}$H NMR analysis of the reaction mixtures for oxidation of Se2Ura (**1a**, 10 mM) with $H_2O_2$ (5 mM) in 67 mM phosphate buffer pH 7.4, at room temperature.

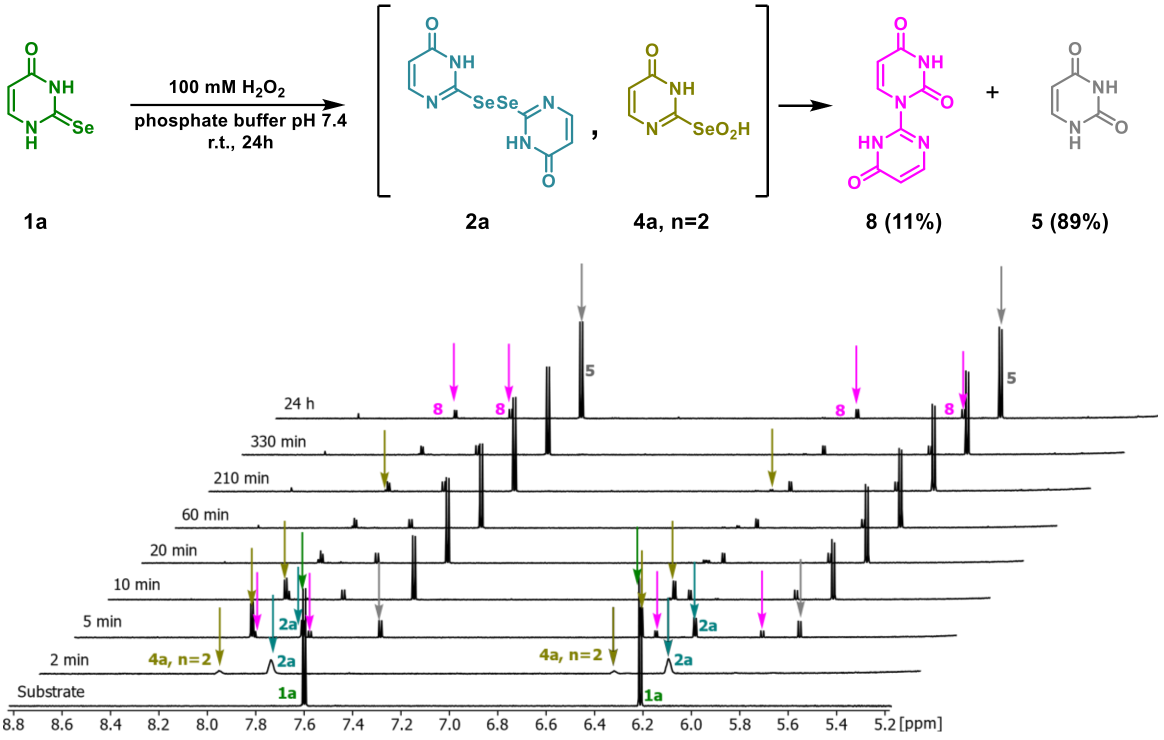


**Figure S25.** $^1H$ NMR analysis of the reaction mixtures for oxidation of Se2Ura (**1a**, 10 mM) with $H_2O_2$ (100 mM) in 67 mM phosphate buffer pH 7.4, at room temperature

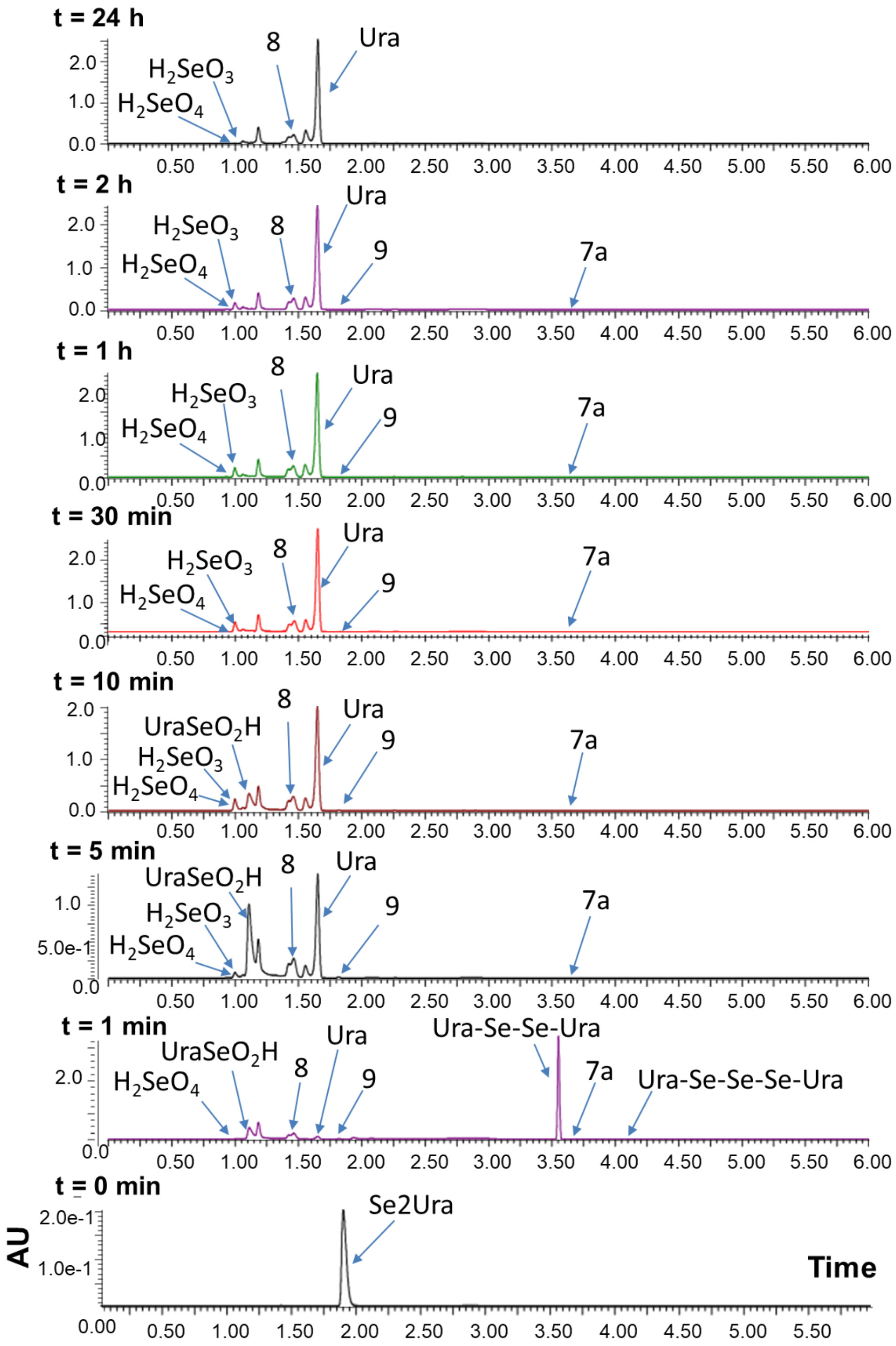


**Figure S26**. UPLC-PDA chromatographic analysis of the reaction mixtures for oxidation of Se2Ura (**1a**, 10 mM) with $H_2O_2$ (100 mM) in 67 mM phosphate buffer pH 7.4 , at room temperature. Inorganic selenic and selenous acids were identified by UPLC-ESI(−)-HRMS and their retention times were determined based on extracted ion chromatograms (EICs) for the ions corresponding to their deprotonated molecules (*m/z* 128.909 and 144.904, respectively).

## 4. MS/MS fragmentation

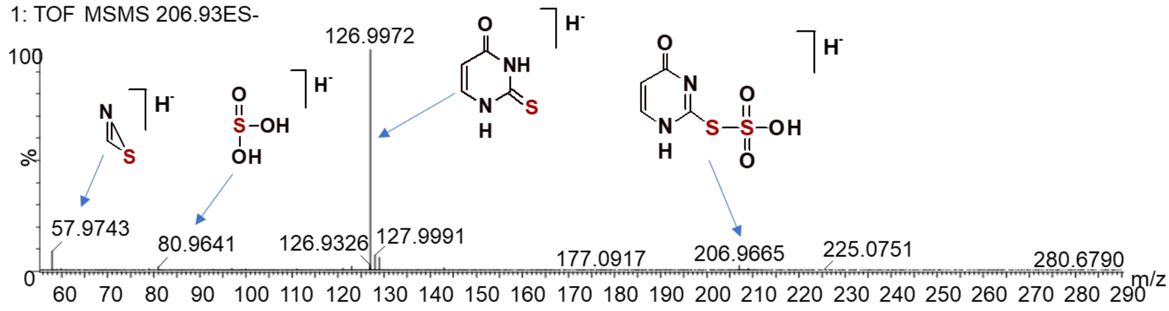


**Figure S29**. Product ion mass spectrum of **10** of MW 208.22 (CE ramped from 15 to 35 eV).

## 5. Time course of the oxidation of S2Ura (1b) by hydrogen peroxide monitored by $^1$H NMR spectroscopy and UPLC-PDA-ESI(−)-HRMS

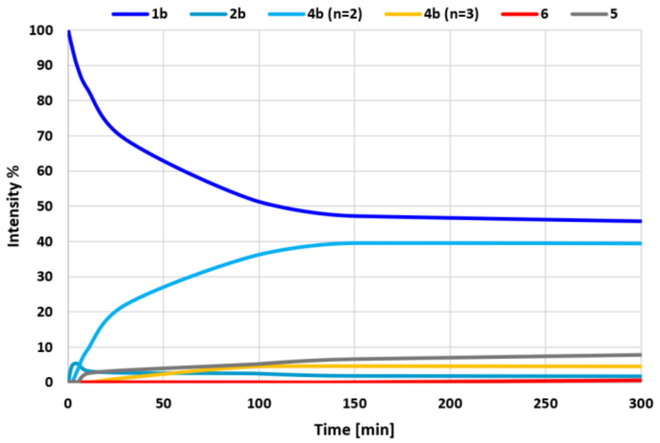


**Figure S28**. The time course of the formation of products for the oxidation reaction of S2Ura (**1b**, 10 mM) with $H_2O_2$ (10 mM) in 67 mM phosphate buffer pH 7.4, r.t.

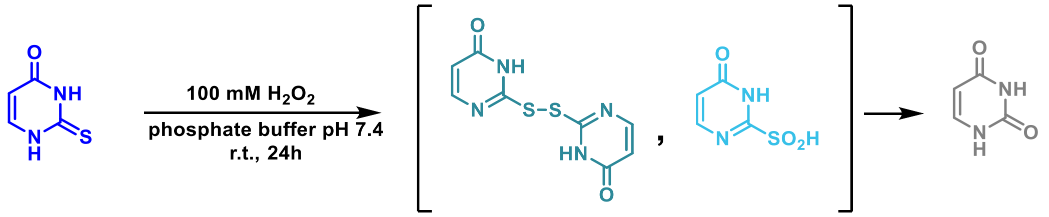


**1b** **2b** **4b, n=2** **100% (5)**

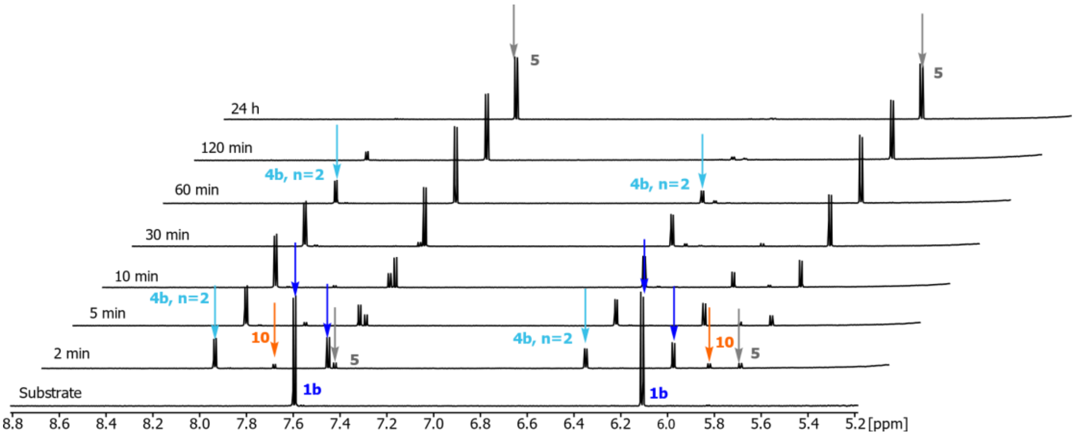


**Figure S29.** $^1$H NMR analysis of the reaction mixtures for oxidation of S2Ura (**1b**, 10 mM) with $H_2O_2$ (100 mM) in 67 mM phosphate buffer pH 7.4, at room temperature (r.t.)

## 6. Analysis of proposed mechanism of formation of 9.

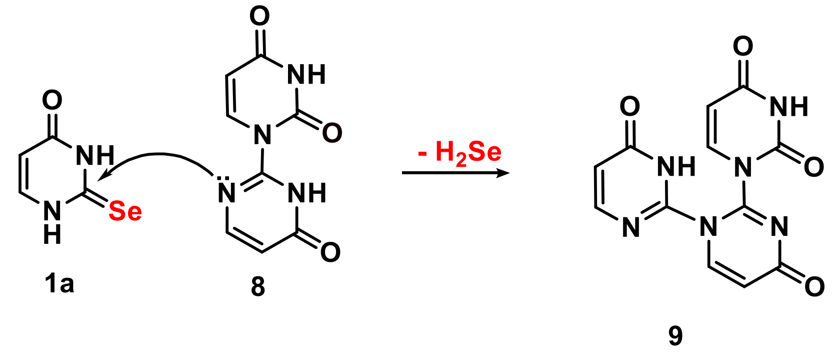


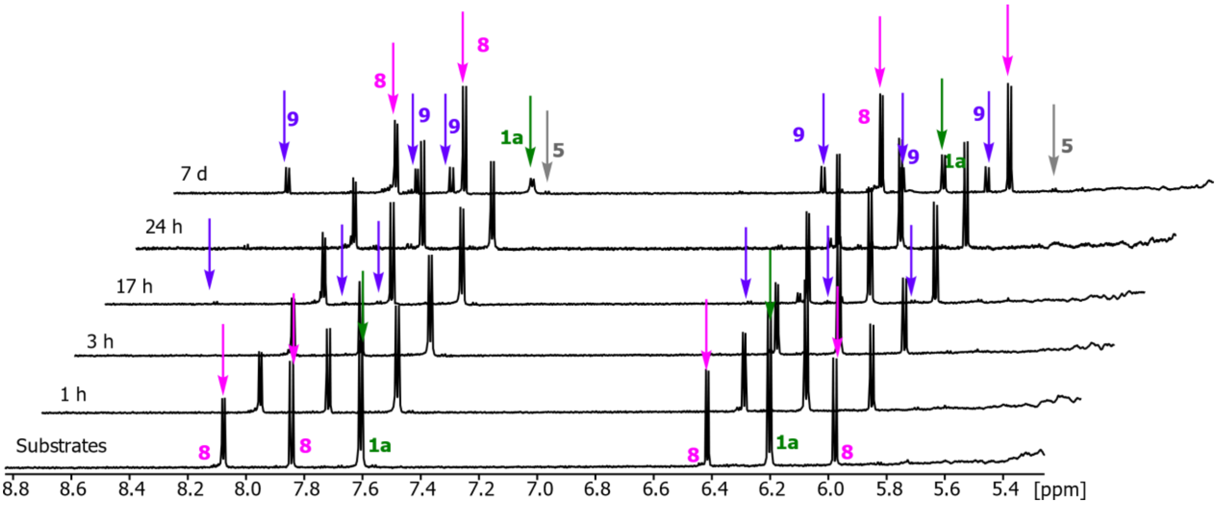

**Fig. S30.** $^1$H NMR analysis (H5 and H6) of the reaction of **1a** (10 mM) with **8** (4 mM) at the 1:0.4 molar ratio, at 67 mM phosphate buffer pH 8, r.t. Traces of compound **9** are seen after 17 and 24 h (signals of H5 and H6 protons are indicated by arrows). After 7 days of incubation the amount of **9** increased to ca. 14%, while the amount of **1a** dropped down to ca. 32%, while **8** was 52% and **5** was 2%.

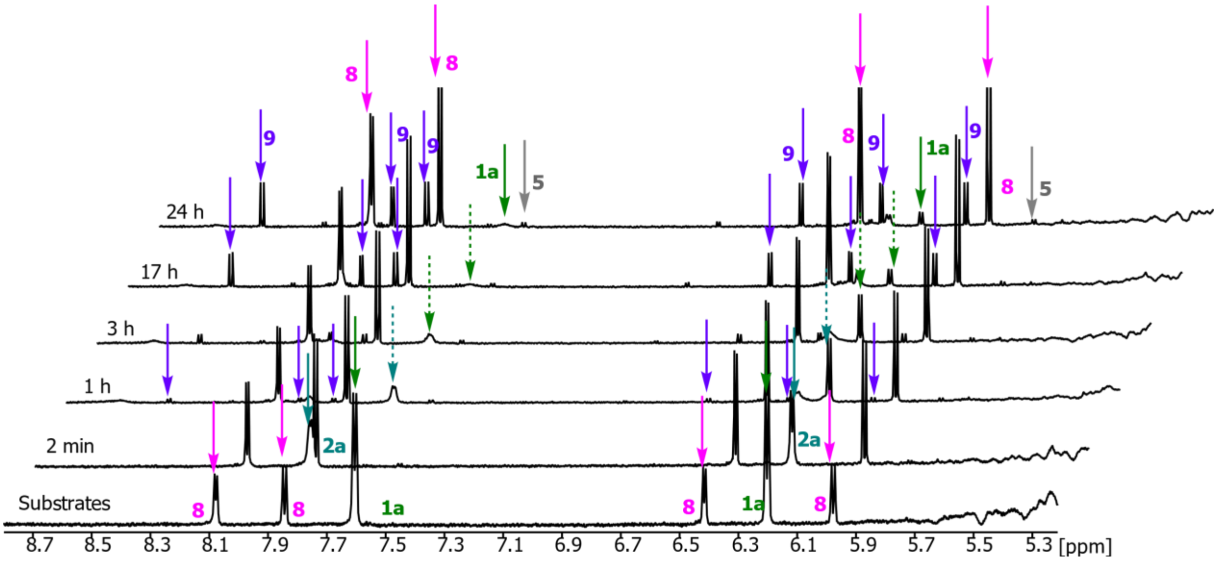


**Fig. S31**. $^1$H NMR analysis (H5 and H6) of the reaction of **8** (4 mM) and **1a** (10 mM) at the 0.4:1 molar ratio, after addition of 0.5 eq. of $H_2O_2$ (5 mM), at 67 mM phosphate buffer pH 8, r.t. Signals of compound **9** are seen after 1 h (see signals of H5 and H6 protons indicated by arrows).
